# The Antidiabetic Activity of Combining the Aqueous Extracts of Vernonia amygdalina Leaves and Tamarindus indica Fruit Pulp in Streptozotocin-Induced Wistar Rats

**DOI:** 10.7759/cureus.46807

**Published:** 2023-10-10

**Authors:** Florian Amel Tekou, Cerile Ypolyte Woumbo, Michel Pegui Kemtsop, Jean Paul Dzoyem, Dieudonné Kuate, David Todem

**Affiliations:** 1 Department of Biochemistry, University of Dschang, Dschang, CMR; 2 Department of Epidemiology and Biostatistics, Michigan State University, East Lansing, USA

**Keywords:** type 2 diabetes mellitus, antioxidant, hypoglycemia, tamrindus indica, vernonia amydalina, diabetes mellitus

## Abstract

Background

Many plants are used to reduce the side effects of diabetes mellitus. These plants (including *Vernonia amygdalina* and *Tamarindus indica*) are rich in phytochemical compounds that have the ability to reduce glycemia and the effect of diabetes-related oxidative stress. In this study, we aimed to investigate the antioxidant and antidiabetic activities of combining *V. amygdalina leaves* and *T. indica* pulp extracts.

Methodology

We prepared a mixture by combining *V. amygdalina* leaves and *T. indica* pulp extracts, and we assessed antioxidant properties via the capacity of both extracts to reduce ferric ions, 2,2-diphenyl-1-picryl-hydrazyl (DPPH) radicals, and hydroxyl radicals. We also assessed antidiabetic properties through the capacity of the extracts’ combination to inhibit alpha-amylase. We evaluated crude fiber, total phenols content (TPC), and total flavonoid content (TFC).

Results

From our findings, the combination at a concentration of 200 μg/mgE showed that a percentage of 55.17±1.2 could reduce DPPH radicals, 0.366±0.012 could scavenge ferric ions, and 0.233±0.0022 could reduce hydroxyl radicals. With regard to secondary metabolites, we obtained 16.96±0.17 mEGA/gE for total phenol content, 1.74±0.045 mECAT/gE for total flavonoid content, and crude fiber content in our combination at 6.87±1%. These results were obtained with a significant difference at the 5% threshold. The extract combination also showed an alpha-amylase inhibitory percentage of 23.56±4.6% at the concentration of 200 μg/mgE. Daily administration of the combination of extracts significantly lowered the fasting blood glucose, triglycerides, total cholesterol, LDL cholesterol, aspartate aminotransferase, alanine aminotransferase, alkaline phosphatase, creatinine, and malondialdehyde. However, there was a significant increase in serum proteins and HDL cholesterol. We did not observe an antagonistic effect between our combination and glybenclamide.

Conclusion

Our formulation, therefore, presents antioxidant and antidiabetic activity and could be used for the management of diabetic patients.

## Introduction

Several factors, including environmental, genetic, and social, are primarily responsible for the health woes of today. Major environmental factors are eating habits and lifestyles, which are full of less sedentary, and the combination of these factors with genetics can lead to cardiovascular diseases such as hypertension, obesity, and diabetes [1]. Diabetes is a disorder resulting from a lack of insulin production or inadequate utilization of insulin by the organism. Type 2 diabetes, directly linked to obesity, accounts for 80% of diabetes cases [2]. The number of adults suffering from diabetes has passed from 108 million in 1980 to 422 million in 2014, and recent estimates indicate more than 435 million people will be diabetic by 2030 if nothing is done [3]. Oxidative stress has long been associated with diabetes as it is responsible for the many deadly complications associated with the disease. Many drugs are produced to treat diabetes; however, in addition to being expensive, these synthetic drugs have serious side effects [4]. Hence, there is an ever-increasing interest in plants and functional foods for diabetes mellitus control. Many studies have already been conducted in this light, and they have revealed that the fruits of *Tamarindus indica* and the leaves of *Vernonia amygdalina* possess bioactive compounds endowed with therapeutic properties. In 2017, Adeoye et al. [5] showed that* V. amygdalina* leaf extracts possessed hypoglycemic activity in alloxan-induced diabetic rats. Mgbeje et al. [6] also showed the leaf fractions of *V. amygdalina* were able to reduce hepatic disorders because of diabetes. Rajesh et al. [7] further revealed the aqueous extract of *T. indica* fruit pulp showed antihyperglycemic potential. Likewise, Razali *et al*. [8] noted that the presence of polyphenols in *T. indica* fruits was responsible for antioxidant activities. In the same line, we conducted the present study to evaluate the antidiabetic and antioxidant properties of the aqueous leaves of *V. amygdalina* and *T. indica* pulp extracts in streptozotocin-induced diabetic Wistar rats.

## Materials and methods

Plant material

We collected *T. indica* fruit samples in the Northern Region of Cameroon and *V. amygdalina* leaves in Dschang (West Cameroon). These samples were identified at the National Herbarium of Cameroon in Yaoundé and sent to the Research Unit of Biochemistry, Medicinal Plants, Food Sciences and Nutrition at the University of Dschang.

Laboratory animals

We obtained four-week-oldWistar rats (males and females) from the Department Animal Centre and allowed them to become accustomed to their new environment for one week. We took care of them in accordance with the guidelines of the OECD [9] and randomly distributed them into eight groups of seven animals (including two controls). The animals were individually housed under controlled temperature (25°C) and lighting (12:12-hour light-dark cycle) and had free access to water and diet. We fed the test groups and the positive control with a high-fat-high-sucrose diet (17.6% fat and 7% sucrose-enriched), whereas the negative control received a basal diet. The high-fat-high-sucrose diet to induce obesity was conducted for 12 weeks and aimed for a body mass index greater than 0.68 g/cm^2^ [10]. These obese animals were administered by an intraperitoneal route, with a single dose of 45 mg/kg of streptozotocin. Three days after the injection of streptozotocin, we considered the animals with a fasting blood glucose concentration higher than 200 g/l as diabetic [11] and used them for the treatment (21 days).

Chemicals

We purchased alpha-amylase, streptozotocin, Folin-Ciocalteu reagent, and DPPH from Sigma-Aldrich, Germany, and we prepared the extracts and reagents on the day of the experiments. We gave streptozotocin as a disease inducer to the rats intraperitoneally (IP), and we administered the extracts and the extract mixture by oral gavage.

Extraction

We obtained an aqueous extract of *V. amygdalina* leaves and *T. indica* pulps according to the method described by Razali et al. [12] by maceration of powders (10 g) of samples in 100 ml of water for 24 hours with gentle stirring. After which, the mixtures were filtered using a Whatman Grade 1 filter paper (Cytiva, MA). We dried the resulting filtrates at 45°C in an air oven to obtain the aqueous extract. We then weighed the extracts to calculate the yield and made a mixture of extracts using one-third extract of *V. amygdalina* leaves and two-thirds *T. indica*; the mixture and extracts were stored in the freezer at -4°C for later use.

Yield (%) = Mass of extract × 100 /Mass of powder

Evaluation of the phytochemical composition

We screened the different extracts and mixtures for the presence of various phytoconstituents like alkaloids, flavonoids, steroids, tannins, glycosides, triterpenoids, and saponins. We used the method described by the Association of Official Analytical Chemists (AOAC) [13] to determine the macro- and microelements.

Total phenol content

We determined the total phenol content of the extracts by the spectrophotometric method using the Folin-Ciocalteu reagent, as described by Dohou et al. [14]. The experimental protocol is summarized as follows: in a test tube, we introduced 10 μL of an extract solution, with a concentration of 2 mg/mL. Then, we successively added 1.39 mL of distilled water, and 0.2 mL of Folin-Ciocalteu’s reagent. After three min of rest, we added 400 μL of sodium carbonate (Na_2_CO_3_, 20%). The tubes were vortexed and incubated for 20 min in a 40°C water bath, and absorbance was read against a blank at 760 nm using a spectrophotometer. We drew the calibration curve using a prepared aqueous solution of gallic acid (0.2 g/L).

Flavonoid content

We determined the flavonoid content according to the method described by Boizot et al. [15] and summarized it as follows: 100 μL of extract with a concentration of 2 mg/mL, 1.4 mL of distilled water, and 30 μL of a 5% sodium nitrite solution were successively introduced into a tube of NaNO_2_. After five min, we added 200 μL of a 10% aluminum trichloride (AlCl_3_) solution, followed by 200 μL of 10% concentrated sodium hydroxide (NaOH) solution and 240 μL of distilled water. We stirred the solution using a vortex and read the absorbance at 510 nm.

Crude fiber content

We analyzed aqueous extracts of different samples for crude fiber content using a ceramic fiber filter as described by AOAC [13]. These extracts and powders were first treated to remove lipids using hexane (24 hours soaking of 6 g of extracts and powders in 30 ml of hexane with gentle stirring). Briefly, we added 100 ml of 1.25% H_2_SO_4_ to 1 g of lipid-free powder in a round bottom flask and boiled it under reflux for 30 min. The hot solution was quickly filtered under suction. We then washed the insoluble matter several times with hot distilled water until it was acid-free and quantitatively transferred it to the flask. Next, we added 100 ml of hot 1.25% sodium hydroxide (NaOH) solution and boiled the mixture again under reflux for 30 min before it was quickly filtered under suction. We washed the soluble residue with boiling water until it was base-free. We then dried it to a constant weight in the oven at 105°C, cooled it in a desiccator, and weighed it. We incinerated the weighed sample (C1) in a muffle furnace at 300^o^C for about 2 hr, cooled it in the desiccator, and reweighed it (C2). The loss in weight of the sample during incineration was given by C1-C2, whereas the crude fiber content was expressed as follows:

Crude fiber (%) = (C1-C2) × 100/weight of original sample

Antioxidant and anti-radical parameters

Determination of Percentage Inhibition of the DPPH Radical

We evaluated the antioxidant activity of the different extracts using DPPH (2,2-diphenyl-1-picryl1-hydrazyl), as described by Mensor [16]. We compared the ability to trap the hydroxyl radical of the extract with that of vitamin C. We prepared the extracts at different concentrations (in μg/mL). We introduced extracts (100 μL) into the tubes. We then introduced 900 μL of DPPH solution into the first two tubes and pure ethanol (900 μL) into the tubes. We then brought everything to room temperature and placed it in the dark for 30 min. We made a reading by measuring the absorbance at 517 nm via a spectrophotometer. For each concentration, we repeated the test twice, and we prepared the blanks under the same conditions. The result is expressed as follows:

% of inhibition= 100 x (control absorbance-sample absorbance)/control absorbance

Determination of Percentage Inhibition of the OH Radical

We determined the ability of the extracts and mixture to trap hydroxyl radicals using the method described by Halliwell et al. [17] in comparison with vitamin C. We generated hydroxyl radicals by the Fe^3+^-ascorbate-EDTA-H_2_O_2_ system (Fenton reaction). The assay is based on the quantification of the 2-deoxy-d-ribose degradation product, which forms a pink chromogen upon heating with TBA at low pH. The reaction mixture contained 0.8 mL of phosphate buffer solution (50 mmol L-1, pH 7.4), 0.2 mL of extractives/standard at diﬀerent concentrations (12.5-150 μg/mL), 0.2 mL of EDTA (1.04 mmol L-1), 0.2 mL of FeCl_3_ (1 mmol L-1), and 0.2 mL of 2-deoxy-d-ribose (28 mmol L-1). We kept the mixture in a water bath at 37°C, and we began the reaction by adding 0.2 mL of ascorbic acid, AA (2 mmol.L-1), and 0.2 mL of H_2_O_2_ (10 mmol L-1). After incubation at 37°C for 1 h, we added 1.5 mL of cold thiobarbituric acid, TBA (10 g L-1), to the reaction mixture, followed by 1.5 mL of HCl (25 %). The mixture was heated at 100°C for 15 min and then cooled down with water. The absorbance of the solution was measured at 532 nm with a spectrophotometer. We evaluated the hydroxyl radical scavenging capacity with the inhibition of a percentage of 2-deoxy-d-ribose oxidation on hydroxyl radicals. We calculated the percentage of hydroxyl radical scavenging activity according to the following formula:

% hydroxyl radical scavenging activity = (A0- (A1-A2) × 100/A0

Where A0 is the absorbance of the control without a sample, A1 is the absorbance after adding the sample and 2-deoxy-D-ribose, and A2 is the absorbance of the sample without 2-deoxy-d-ribose.

Determination of Antioxidant Activity by the FRAP Method

We evaluated the ferric ions’ scavenging capacity of the extracts and extracts mixture according to the FRAP method, as described by Oyaizu [18]. In test tubes previously containing 100 μL of extract solution at different concentrations (in μg/mL) prepared in the hydroethanol solution (25:75), we added 500 μL of a phosphate buffer solution (0.2 M pH 6.6), followed by 500 μl of aqueous solution of potassium hexacyanoferrate (K_3_Fe (CN)_6_) 1%. We incubated the mixture for 30 min at 50°C in a water bath. Afterward, we added 500 μl of 10% trichloroacetic acid solution and centrifuged the mixture at 3,000 rpm for 10 min. We prepared a standard metal reducer (vitamin C) under the same conditions to compare the reducing power of the different extracts. The blank consisted of all reagents except the extracts. We read the absorbance of the reaction mixture at 700 nm against that of the blank and conducted the tests in triplicate. An increase in absorbance corresponded to an increase in the reducing power of the extracts tested. The result is expressed in mg/ml of extract.

In Vitro Inhibition of Alpha-Amylase

We conducted the evaluation of the in vitro α-amylase inhibitory activity of extracts using the method described by Bernfeld [19]. In brief, we reacted 0.02-4 µL of the different extracts with 200 µL of α-amylase enzyme and 100 µL of 2 mM of phosphate buffer (pH 6.9). After 20 min preincubation, we added 100 µL of 1% starch solution. We performed the same for the controls. After 5 min of incubation, we added 500 µL of dinitrosalicylic acid reagent to both the control and the test. They were kept in a boiling water bath for 5 min, at which point we recorded the absorbance at 540 nm using a spectrophotometer and calculated the percentage inhibition of α-amylase enzyme using the following formula:

% Inhibition = ([Control-Test]/Control) × 100

Treatment of rats with extracts and mixture 

We randomly divided the rats into eight groups of seven rats: group I received an aqueous extract of* Tamarinus indica* (500 mg/kg), group II received aqueous extract of *V. amygdalina* (500 mg/kg), group III received a mixture of *V. amygdalina* and *T. indica* (500 mg/kg), group IV received the mixture of *V. amygdalina* and *T. indica* (250 mg/kg), group V received the mixture of *V. amygdalina* and *T. indica* (500 mg/kg) in association with glybenclamide (80 mg/kg), group VI received glybenclamide (80 mg/kg), and group VII and group VIII received saline and served as the normal and positive control. 

Animal sacrifice

Twenty-four hours after the last administration of extracts and formulation, we sacrificed the animals under light anesthesia using ketamine (administered by the intraperitoneal route at 10 mg/kg). Dissecting instruments were washed with 5% nitric acid and rinsed several times with distilled water before use.

Serum preparation

After sacrificing the rats, we collected the blood of the different animals and then centrifuged it at 3,500 rpm for 30 min. We collected the filtrate obtained using a micropipette and introduced it into labelled Eppendorf tubes before being stored at -18°C for subsequent assay of the biochemical parameters of interest.

Acute toxicity studies

We evaluated the acute toxicity of the extracts and the mixture according to the protocol described by OCDE [20] regarding the assessment of the acute toxicity of chemical products.

Biological parameters

We measured blood glucose level (expressed in mg/dl) using 5-10 µL of blood obtained from the tail tip cut after overnight fasting (8 hrs) using a portable glucometer (Accu-Chek) every five days during the treatment.

We determined the lipid profile using colorimetric methods (MONLAB kits; Monlab, Barcelona, Spain) using the standard protocols described by Fossati et al. [21] for triglycerides and Trinder [22] for total cholesterol. Drawing on Huang et al. [23], we estimated the HDL cholesterol and LDL cholesterol using the formula established by Friedewal et al. [24].

We also used calorimetric methods (IMESCO kits; BIOLABO S.A.S., Les Hautes Rives, France) to determine the serum level of alanine aminotransferase, aspartate aminotransferase, and alkaline phosphatase, and we utilized the standard protocol described by Lowry et al. [25] to determine total protein and that by Bartels et al. [26] for creatinine. We also used Yagi’s method [27] to determine the total level of malondialdehyde (MDA) in the serum of the different animals.

Statistical analysis

We expressed data ​​as the mean±standard error of the mean. After the analysis of variance, we conducted the comparison of means between different groups by the Waller-Duncan test using SPSS software (version 26.0; IBM SPSS Statistics for Windows, Armonk, NY). P values ​​<0.05 were considered statistically significant.

## Results

Phytochemical composition, antioxidants, and antiradical activities

Phytochemical Composition of Extracts and Mixture

Table 1 shows the results obtained for the qualitative analysis of components present in each extract and mixture. It appears the mixture of extracts contains many compounds that can act to aid the management of diabetes. We also note that the mixture contains tannins, which are not present in *T. indica* pulp extracts.

**Table 1:** Phytochemical analysis. +: Presence of compound; -: absence of compound.

	T. indica	V. amygdalina	Mixture of extracts (formulation)
Alkaloids	+	+	+
Phenols	+	+	+
Flavonoids	+	+	+
Sterols	-	-	-
Triterpenoids	+	+	+
Tannins	-	+	+
Saponins	+	+	+

Total phenol content

**Figure 1:**
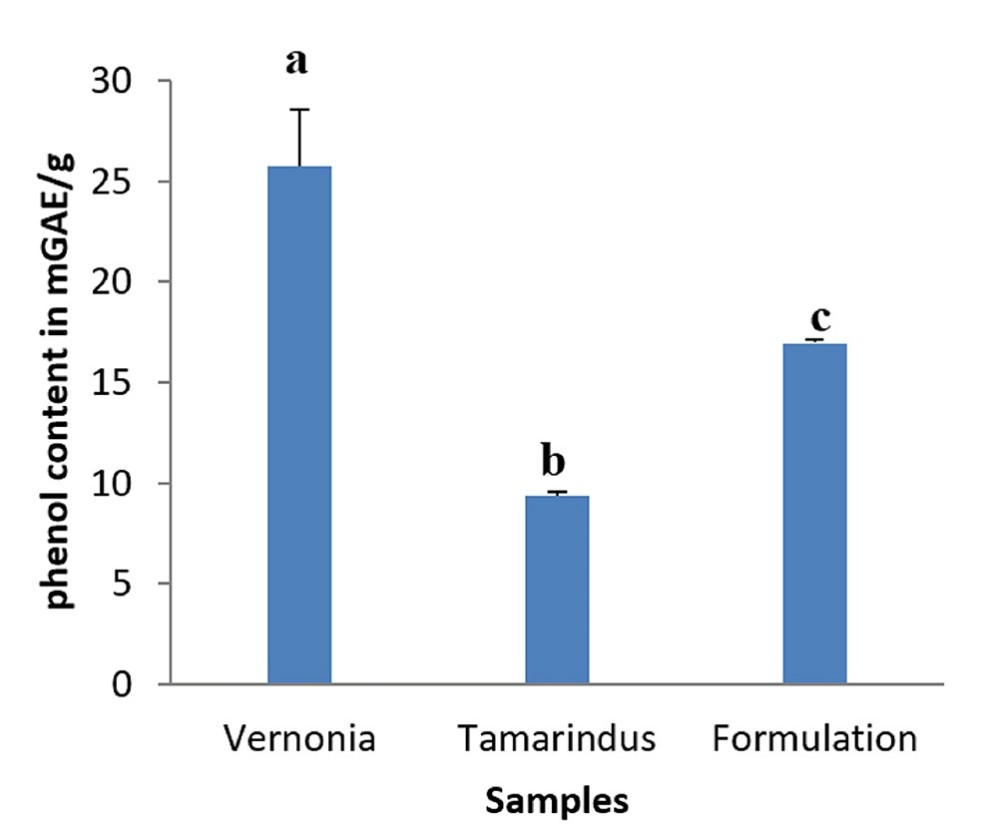
Total phenol content (TPT) of extracts and mixture. a, b, and c: Bands with different letters are significantly different at p<0.05.

Figure 1 shows the total phenol contents of *T. indica* pulp extract, *V. amygdalina* leaves extract, and the mixture of the two extracts, expressed in mg of gallic acid equivalent/g of different samples. We observed that the total phenols content mixture was about 16.9±0, 17 mGAE/g.

Flavonoid content

**Figure 2:**
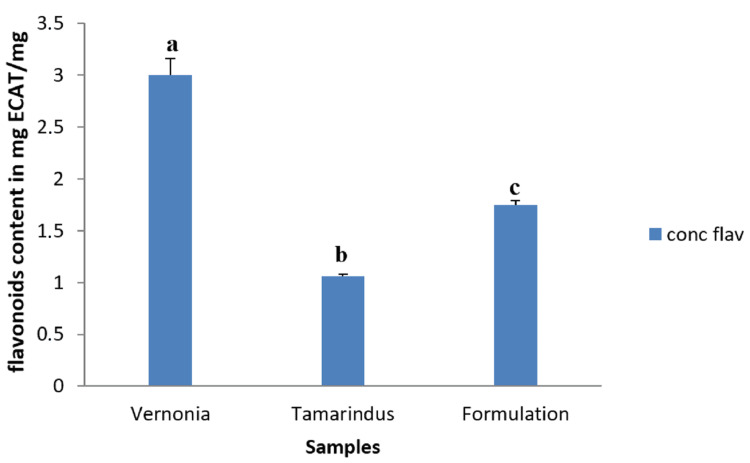
Total flavonoid (TFT) content of extracts and formulation. a, b, and c: Bands with different letters are significantly different at p<0.05.

Figure 2 shows the flavonoid content of our different samples expressed in mg equivalent of catechin/mg of sample. It appears that the flavonoid content varied from 1.05±0.02 for *T. indica* to 3.00±0.15 for *V. amygdalina*. The mixture presented 1.76 ±0.04 mECAT/gE as flavonoid content.

Crude fiber content

**Figure 3:**
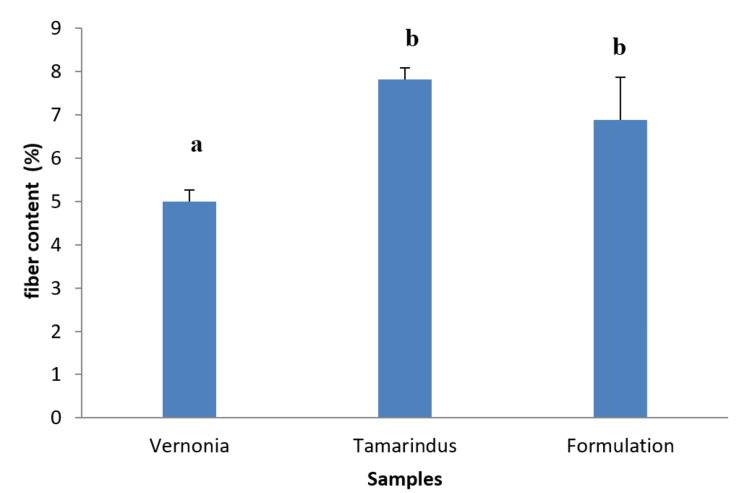
Crude fiber content of extracts and mixture. a, b, and c: Bands with different letters are significantly different at p<0.05.

Figure 3 shows the results obtained for crude fiber content; it appears the mixture of extracts contained 6.76±0.1% crude fiber content, *V. amygdalina* leaves contained 5.33±0.3% crude fiber content, and *T. indica *contained 7.48±0.7% crude fiber content.

DPPH radical scavenging capacity

**Figure 4:**
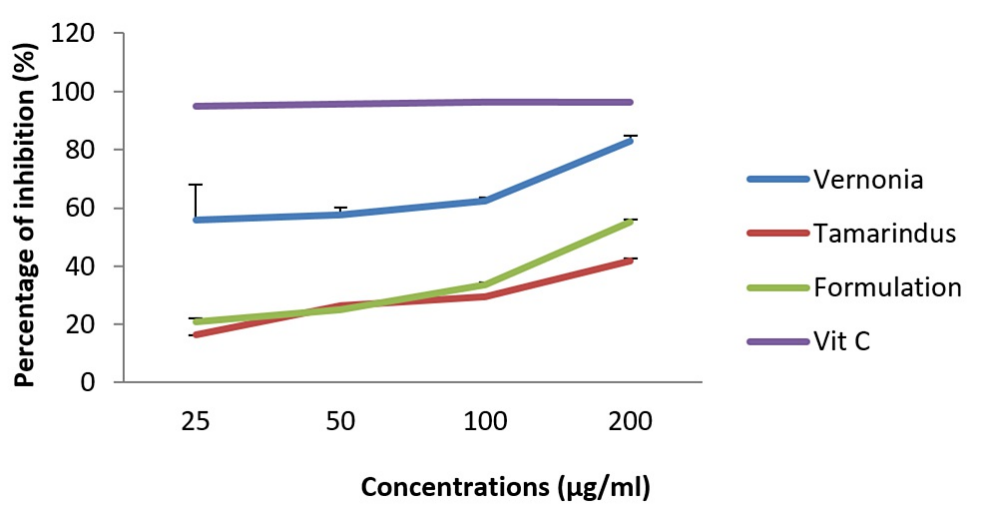
Anti-radical activity of extracts and mixture at different concentrations compared to vitamin C.

Figure 4 below shows the ability of our different samples to inhibit DPPH radicals compared to a conventional inhibitor (i.e., vitamin C). It appeared the mixture was able to reduce DPPH radicals in a dose-dependent manner. At 200 µg/mL, the formulation presents a reduction of 55.17±0.78%, but this remains less than the reducing power of vitamin C.

Iron-reducing capacity

**Figure 5:**
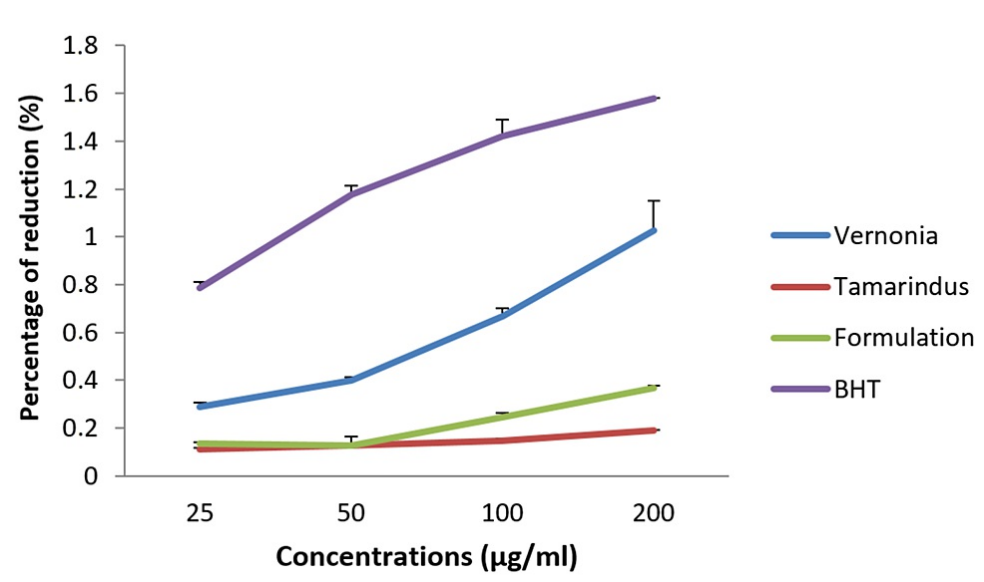
Reducing power of extracts and mixture compared to BHT. BHT: Biturylhydroxytomuene.

We also evaluated the ability of our samples to reduce iron in comparison with butylated hydroxytoluene (BHT). Figure 5 below shows the capacity of the extracts and mixture of extracts to reduce iron ions. It appears that, depending on the dose, the mixture of extracts was able to reduce ferric ions. At a dose of 200 µg/ml, the reduction power of the mixture of extracts was 0.366±0.012%, less than the reducing power of our control, BHT.

Hydroxyl radical scavenging ability

**Figure 6:**
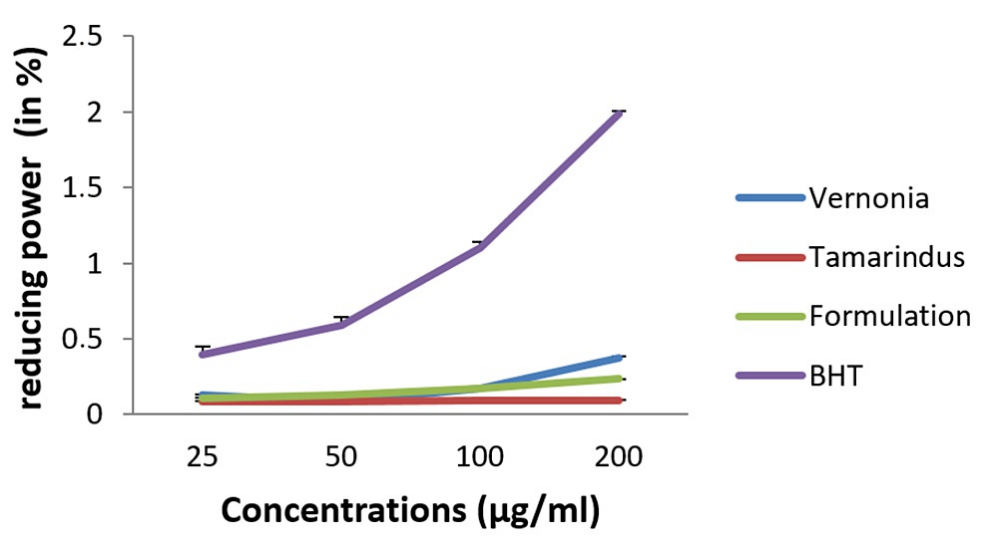
OH-reducing power of extracts and the formulation in comparison with BHT.

Figure 6 shows the OH-reducing power of extracts and the mixture of extracts at different concentrations. We also observed that, when mixing extracts at a dose of 200 µg/mL, we obtained 0.233±0.0022% as a percentage of reduction of OH radicals.

Alpha-amylase inhibitory activity of extracts and mixture

**Figure 7:**
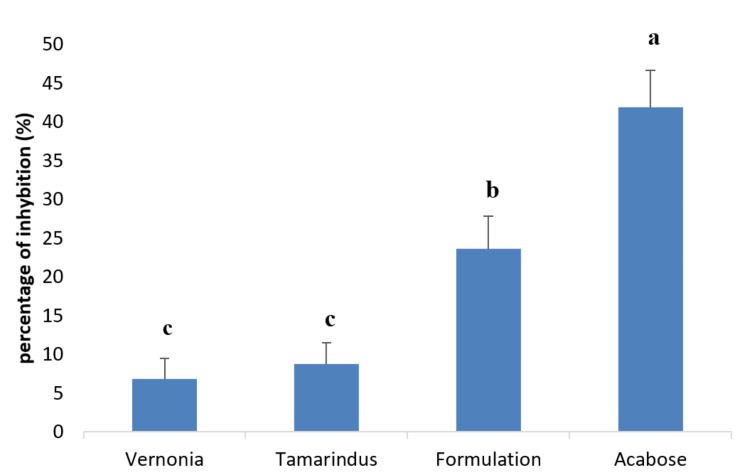
Effect of extracts and mixture of extracts in vitro on alpha-amylase activity, in comparison with acarbose. a, b, and c: Bands with different letters are significantly different at p<0.05.

Figure 7 shows the ability of our formulation to inhibit alpha-amylase at the concentration of 100 µg/ml in comparison with acarbose. It appears that the percentage of inhibition of our mixture is less than that of Acarbose, but the mixture of extracts better inhibited alpha-amylase than the different extracts taken separately.

Effect of extracts and mixture on some biochemical parameters of diabetic rats

Administered at doses up to 2,000 mg/kg orally, our mixture did not produce any signs of toxicity. Hence, for further studies, 250 and 500 mg/kg doses of the mixture were selected. The effect of the extracts and the mixture on the blood sugar of the animals during the treatment was evaluated.

Table 2 shows the evolution of the glycemia (in mg/dl) of diabetic rats during the three-week period of treatment. There is a significant reduction of glycemia in animals receiving the mixture of extracts at the concentration of 500 mg/kg. We also noticed a reduction of glycemia in animals receiving both the mixture of extracts and glybenclamide at 80 mg/kg.

**Table 2:** Changes in blood sugar (mg/mL) during treatment. T.ind: Tamarindus indica; V.am: Vernonia amygdalina; T+V 500: formulatin at 500 mg/kg; T+V 250: formulation at 250 mg/kg; T+V+Glyb: formulation and glybenclamide; Glyb: glybenclamide; C(+): positive control; C(-): negative control.

	Day 0	Day 5	Day 10	Day 15	Day 20	Variation (D0-D21)
T.ind	309±22	219±32	208±8.6	140±13	135±6.4	-174
V.am	359±39	267±21	231±31.4	194±20	197±31	-162
T+V 500	306.4±14.8	226.8±17.9	190.6±21.71	144.6±29	135.8±29	-181
T+V 250	280.25±21.83	242.25±21.68	221.5±17.17	192.25±15.64	167±11.5	-113
T+V+Glyb	267±8	235±7.08	191±5.1	175±3.24	116±8.3	-151
Glyb	354.33±4.26	267.66±13.08	206±6.9	154±5.33	138±9.2	-216
C(+)	357.33±12.75	385.33±9.54	396±3.24	382.66±10.63	392±12.86	+35
C(-)	70.2±5.26	81±7.72	85.8±5.9	81.8±4.8	70±3.68	-0.2

Antihyperglycemic potential of extracts and mixture: oral glucose tolerance test (OGTT)

**Figure 8:**
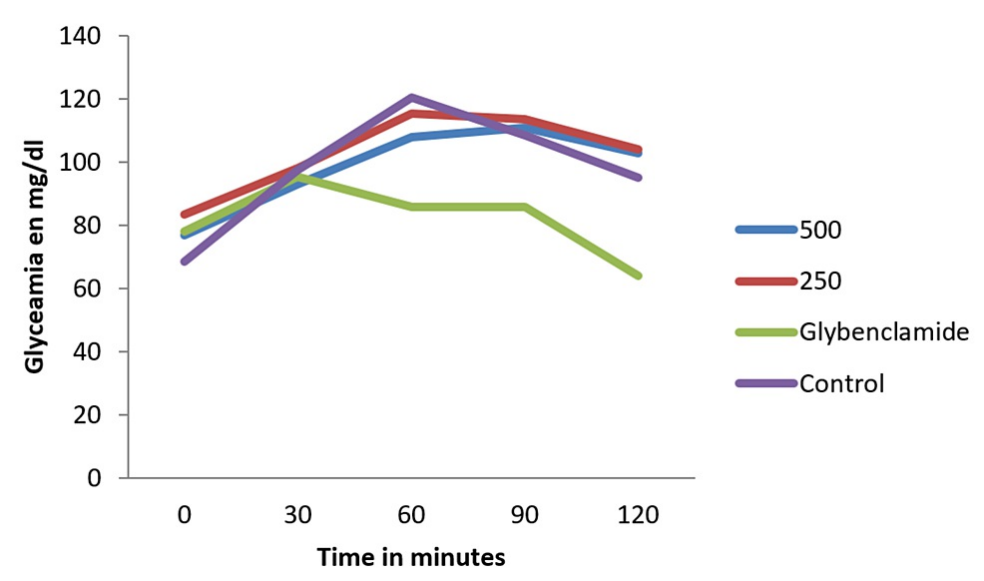
Changes in blood glucose during oral glucose tolerance test (mg/dl).

Figure 8 shows the results obtained for the oral glucose tolerance test. It appears the glycemic peak of rats that took our formulation was lower than those of the control (receiving glucose only). Even if this peak was higher than those who took glybenclamide, our mixture was able to promote an antihyperglycemic activity in a dose-dependent manner.

Effect of the treatments on body weight

Table 3 presents the differences observed in the body weight of the diabetic rats and controls during the treatment. Generally, we observed a weight reduction in all diabetic rats. Moreover, we noticed that this reduction was more important in the positive control than in other groups. It also appears that the reduction obtained for rats receiving the mixture was not different from those receiving the reference drug. In combination with glibenclamide, the mixture presented the highest reduction in body weight.

**Table 3:** Changes in the body weight of diabetic rats and controls during the treatment. T.ind: Tamarindus indica; V.am: Vernonia amygdalina; T+V 500: formulation at 500 mg/kg; T+V 250: formulation at 250mg/kg; T+V+Glyb: formulation and glybenclamide; Glyb: glybenclamide; C(+): positive control; C(-): negative control.

	Day 0 (in g)	Day 10 (in g)	Day 20 (in g)
T.ind	229±1.22	220±1.8	214±1.4
V.am	231±1.8	216±4.4	212±11.5
T+V 500	248±12.5	227±17.4	215±1.4
T+V 250	235±10.8	227±15.8	216±14
T+V+Glyb	240±10.6	223±4.9	197±8.3
Glyb	257±11.57	218±9.9	191±2.12
C(+)	233±2.54	197±11.6	178±3.2
C(-)	244±18.68	264±12.87	300±11.9

Effect of the treatment on the lipid profile

Table 4 depicts the serum concentration of triglyceride, total cholesterol, HDL, and LDL cholesterol of animals after 21 days of treatment. We observed that the mixture was able to reduce the serum levels of triglycerides better than the different extracts. With regard to HDL cholesterol, it appears the highest increase in the serum level of HDL cholesterol was obtained in animals that received our mixture in combination with glybenclamide.

**Table 4:** Serum lipid of animals after the treatment. T.ind: Tamarindus indica; V.am: Vernonia amygdalina; T+V 500: formulation at 500 mg/kg; T+V 250: formulation at 250 mg/kg; T+V+Gly: formulation and glybenclamide; Glyb: glybenclamide; C(+): positive control; C(-): negative control; a, b, and c: values with different letters in the same column are significantly different at p<0.05.

	CH-total (mg /dl)	TAG (mg /dl)	HDL (mg /dl)	LDL (mg /dl)
T.ind	108.42±9.34^a^	52.68±4.6^b^	31.76±1.25^a^	23.97±6.4^a^
V.am	108.71±2.75^a^	88.17±8.6^c^	30.81±0.95^a^	14.37±5.7^a^
T+V 500	104.21±2.37^a^	39.85±10.8^a^	29.1±2.19^a^	30.75±5.95^a^
T+V 250	100.07±6.18^a^	48.38±12^b^	29.98±2.7^a^	21.69±3.8^a^
T+V+Gly	122.96±4.29^b^	47.67±7.8^b^	32.26±1.53^a^	43.02±7.6^a^
Glyb	97.8±2.7^a^	26.16±6.4^a^	30.53±1.08^a^	41.13±9.2^a^
C(+)	167.11±3.8^c^	23.65±1.3^a^	26.79±1.05^b^	116.66±3.03^b^
C(-)	87.51±5.2^a^	30.43±2.4^a^	40.57±3.1^c^	16.5±4.1^a^

Effect of the treatment on the serum level of transaminase, alkaline phosphatase, and total protein

**Figure 9:**
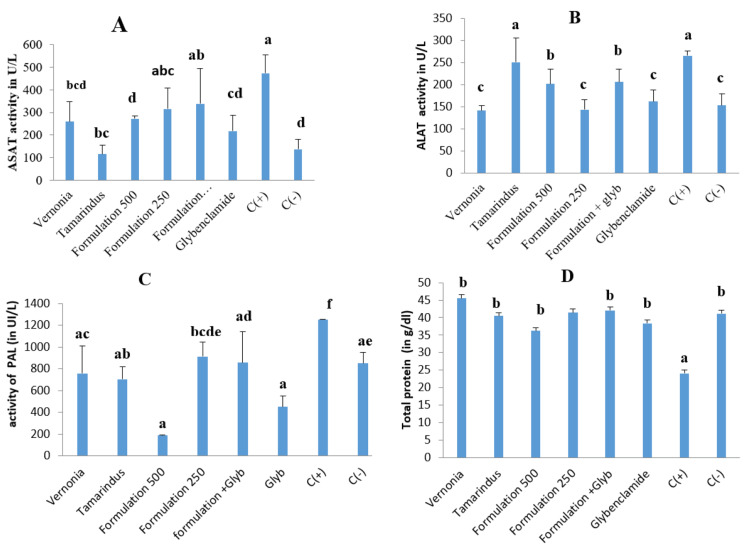
Serum level of transaminases, alkaline phosphatase, and total proteins of animals after the treatment. ALAT: alanine aminotransferase; ASAT: aspartate aminotransferase; PAL: alkaline phosphatase. C(+): positive control; C(-): negative control. a, b, c, d, and e: bands with different letters are significantly different at p<0.05.

Figure 9 (A, B, C, and D) shows the serum level of alanine aminotransferase (ALAT), aspartate aminotransferase (ASAT), alkaline phosphatase (PAL), and total proteins of animals treated with aqueous extracts, the mixture of extracts, glybenclamide, and the combination of our mixture and glybenclamide. Our mixture is able to reduce the serum level of ASAT, ALAT, and PAL, but the combination with the reference drug does not induce any significant changes in the enzyme activity.

Effect of the treatment on the serum level of malondialdehyde

**Figure 10:**
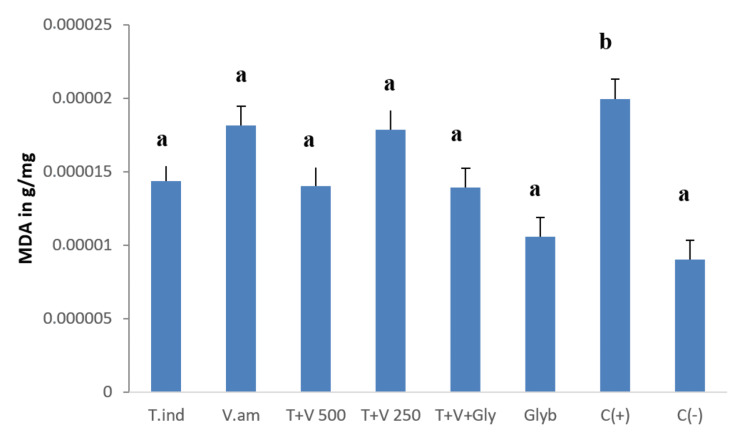
Serum levels of malondialdehyde in the different groups of rats. T.ind: Tamarindus indica; V.am: Vernonia amygdalina; T+V 500: formulation at 500mg/kg; T+V 250: mixture at 250mg/kg; T+V+Gly: formulation and glybenclamide; Glyb: glybenclamide; C(+): positive control; C(-): negative control. a and b: Bands with different letters are significantly different at p<0.05.

Figure 10 presents the serum level of malondialdehyde (MDA) of rats being treated with extracts, our formulation, glybenclamide, and the combination of our formulation and glybenclamide. We can easily observe that our formulation at the dose of 500 mg/kg is able to induce a significant reduction of the serum level of MDA. The combination of our formulation and glybenclamide does not induce a significant change in the serum MDA level of rats.

Effect of the treatment on the urine and serum level of creatinine

**Figure 11:**
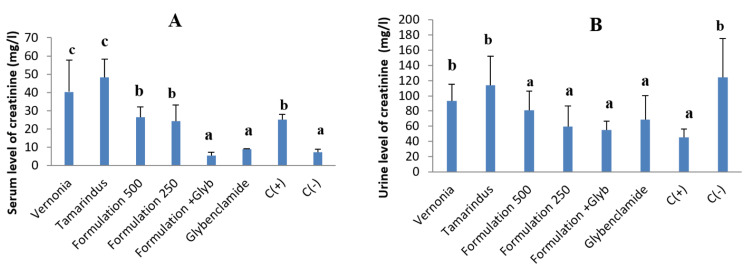
Serum and urine level of creatinine of animals after the treatment. C(+): Positive control; C(-): negative control. a, b, and c: Bands with different letters are significantly different at p<0.05.

Figure 11 (A and B) displays the serum and urine levels of creatinine in animals receiving extracts and our formulation. We observed that animals receiving our formulation presented a significant reduction in the serum level of creatinine and a significant increase in urine creatinine. These observations are more important when combining our formulation with glybenclamide.

## Discussion

Many of the positive effects of plant and functional foods are often attributed to the presence of phenolic compounds. In the case of diabetes, phenolic compounds (as flavonoid compounds) exhibit antioxidant properties that can reduce the complications that often occur in diabetic patients [28]. The phenol content obtained can be attributed to many factors, such as the method of extraction and solvent use. In fact, water used for extraction has the ability to break down biological membranes and share bioactive compounds in the solvent [29]. These results corroborate those of Bourgou et al. [30] who showed that water and alcohol are the best solvents for the extraction of phenolic compounds.

With regard to crude fiber content, the combination of extracts showed a low fiber content that can be attributed to the Whatman paper used during extraction, which could limit the dispersion of dietary fiber in the solvent. Tekou et al. [31] obtained similar results when evaluating the crude fiber content of *V. amygdalina* leaf extracts.

The high antioxidant activity of our mixture shown by the three tests (FRAP, DPPH, and OH radicals) can be attributed to the bioactive compounds present in our formulation, such as the phenolic compounds that include many other subclasses [32]. These compounds may exert actions by neutralizing free radicals or transferring electrons or hydrogen atoms to DPPH [33].

The present work shows our mixture was able to reduce the glycemia of diabetic rats alone or in association with glybenclamide. This reduction is associated with the inhibition of pancreatic alpha-amylase, as shown in vitro, or inhibiting the sodium-dependent glucose transporter in rabbit intestinal epithelial cells. It could also be linked to an increase in the sensitivity to insulin by phenolic compounds, as shown by Dembinska-Kiec et al. [34]. These phenolic compounds (as catechins) can reduce insulin resistance by acting as ligands for peroxisome proliferator-activated receptors with a dual alpha/gamma agonist effect [35].

The mechanism by which aqueous extracts and our formulation exert their activity on the lipid profile is not clearly detailed, but, from our findings, our mixture was able to promote a significant increase in the serum level of HDL and significantly lowered the level of LDL cholesterol and triglycerides. These results can be attributed to the presence in our combination of many phenolic compounds able to act as inhibitors of hepatic synthesis of cholesterol. These results corroborate those of Asante et al. [36], who showed that phenolic compounds have the ability to increase serum HDL cholesterol levels.

Many organs in the body can be affected by diabetes complications. Regarding the liver, our combination demonstrated a hepatoprotective effect by significantly decreasing the serum level of transaminase, malondialdehyde, and alkaline phosphatase and significantly increasing the serum level of total proteins. These results can be attributed to bioactive compounds such as phenols that can help in the recovery from hepatic cell injuries because of diabetes by regulating alterations in liver enzymes and protecting cell membrane permeability or avoiding liver cell rupture [37]. These results are similar to those obtained by Rajangam et al. [38]. Regarding the kidneys, we observed an increase in the level of creatinine in the serum and a decrease in urine creatinine. This can be attributed to the nephrotoxicity of streptozotocin [39]. In the present work, the extract combination showed an ability to reduce damage to the kidney, and this was shown by a significant decrease in the serum level of creatinine in comparison with the control group. This result is in accordance with previous works of Woumbo et al. [40], who reported that okra extract induces a significant decrease in serum creatinine in diabetic Wistar rats.

## Conclusions

The aim of the present work was to demonstrate the presence of phenolic compounds, flavonoids, dietary fiber, and in vitro and in vivo antioxidant activities of a mixture made by combining *V. amygdalina* leaves and *T. indica* pulp extracts. The evidence supports the recovery from diabetic damage caused by streptozotocin in Wistar rats. We found that the mixture of these extracts contains many bioactive compounds with in vitro antioxidant activity. In the same way, the mixture acted as an inhibitor of pancreatic amylase and showed in vivo antidiabetic activity in streptozotocin-induced diabetic Wistar rats. Further detailed studies with purified compounds could provide evidence to effectively control diabetes.
